# Comparative Transcriptome Analysis Reveals Key Functions of MiMYB Gene Family in Macadamia Nut Pericarp Formation

**DOI:** 10.3390/ijms25136840

**Published:** 2024-06-21

**Authors:** Qiujin Tan, Xiuju Huan, Zhenzhen Pan, Xiaozhou Yang, Yuanrong Wei, Chunheng Zhou, Wenlin Wang, Lifeng Wang

**Affiliations:** 1 Guangxi South Subtropical Agricultural Research Institute, Longzhou 532415, China; tqiujin110@gxaas.net (Q.T.);; 2 Rubber Research Institute, Chinese Academy of Tropical Agricultural Sciences, Haikou 571101, China; 3 Guangxi Academy of Agricultural Sciences, Nanning 530007, China

**Keywords:** macadamia nut, pericarp, transcriptome, MiMYB, flavonoid

## Abstract

Macadamia nuts are one of the most important economic food items in the world. Pericarp thickness and flavonoid composition are the key quality traits of Macadamia nuts, but the underlying mechanism of pericarp formation is still unknown. In this study, three varieties with significantly different pericarp thicknesses, namely, A38, Guire No.1, and HAES 900, at the same stage of maturity, were used for transcriptome analysis, and the results showed that there were significant differences in their gene expression profile. A total of 3837 new genes were discovered, of which 1532 were functionally annotated. The GO, COG, and KEGG analysis showed that the main categories in which there were significant differences were flavonoid biosynthesis, phenylpropanoid biosynthesis, and the cutin, suberine, and wax biosynthesis pathways. Furthermore, 63 MiMYB transcription factors were identified, and 56 R2R3-MYB transcription factors were clustered into different subgroups compared with those in Arabidopsis R2R3-MYB. Among them, the S4, S6, and S7 subgroups were involved in flavonoid biosynthesis and pericarp formation. A total of 14 *MiMYB*s’ gene expression were verified by RT-qPCR analysis. These results provide fundamental knowledge of the pericarp formation regulatory mechanism in macadamia nuts.

## 1. Introduction

Macadamia (*Macadamia integrifolia*) is a perennial evergreen fruit tree of the family Macadamia, which is native to the subtropical rainforest areas of southeastern Queensland and the northeastern coast of New South Wales, Australia [1]. Known as the “Queen of Dried Fruits” and the “King of Nuts”, macadamias are the world’s most popular edible nuts and have become one of the world’s emerging nut industries. China began to commercially introduce macadamias in the 1970s, and they are now mainly distributed in Yunnan, Guangxi, and Guangdong provinces and regions. At present, China is the country with the fastest growth and the largest planting area of macadamias in the world, with more than 330,000 hectares, ranking first in the world, and the output of unhulled fruit is 110,000 tons, ranking fourth in the world. Macadamias comprise three parts: the pericarp, the shell, and the kernel. The pericarp contains a variety of nutrients, such as crude fat, tannins, crude protein, and phenolic acids [2], which have various properties, such as disease resistance, antioxidant effects, and a protective effect on the shell and kernel. However, since the pericarp is prone to oxidation and fermentation when stored in a stack, it is necessary to peel it on the day of harvesting. Machinery suitable for macadamia fruit processing has been developed. Significant progress has been made in the reuse of macadamia pericarp, as large quantities of macadamia pericarp have been separated [3]. Our previous studies found that the nutrient composition of different macadamia germplasm pericarp was significantly different, and the coefficient of variation of inclusions was between 15.75% and 48.33%. The tannin contents range from 1.06 to 2.16% in macadamia nut pericarps. These pericarps can be fermented into organic fertilizer to increase soil nutrients and organic matter. Recent studies have shown that macadamia pericarp color is associated with changes in kernel quality and can be used as a predictor of harvest time. A novel ultrasound-assisted extraction method can effectively extract anti-aging components from the pericarp and improve its economic value. With advances in macadamia separation and processing technology, macadamia pericarp is widely used in high value-added industries, such as cosmetics and organic fertilizers [3]. The main problem facing China’s macadamia industry is the urgent need for new varieties that are easy to process and rich in active ingredients. It is an effective way to solve the problem of industrial demand by screening and creating germplasm with significant differences in macadamia kernel and pericarp through grafting, hybridization, and transgenic technology, then cultivating high-quality varieties.

Recently, research on plant pericarp or peel development and nutrient composition has made rapid progress. For instance, to measure pericarp thickness, a new non-destructive method using extended-focus optical coherence microscopy was developed [4]. In sweetcorn, it was found that auxin (AUX), gibberellin (GA), and brassinosteroid (BR) signal transduction may indirectly mediate PCD to regulate pericarp thickness in the corn variety with a thin pericarp [5]. By using QTL mapping and transcriptome analysis, it was found that AUX/IAA transcription factor and ZIM transcription factor regulate pericarp thickness [6]. In pomegranate, naringenin, pelargonidin, kaempferol, and *CHI* (Pgr25966.1), *F3′5′H* (Pgr26644.1), and *CHS* (Pgr005566.1) genes were related to pericarp’s response to sunburn, and *MYB93* and *MYB111* may be involved in phenylpropanoid and flavonoid biosynthesis by regulating these genes [7]. Combining transcriptome and metabolome analyses, the transcription factors NAC and MYB were found to possibly be involved in major transcriptional regulatory mechanisms [8]. In rice, it was found that R2R3 MYB TF OsKala3 is a common key player for black rice pericarp [9]. In pepper, *CaR2R3-MYB* genes are involved in regulating the synthesis of capsaicin and dihydrocapsaicin [10,11]. In kiwifruit, *AcMYB10* with *AcbHLH42* may strongly activate anthocyanin biosynthesis by activating the transcription of *AcLDOX* and *AcF3GT* [12]. AcMYB123 and AcbHLH42 are the components involved in the spatiotemporal regulation of anthocyanin biosynthesis, specifically in the inner pericarp of kiwifruit [13]. Cytokinin is involved in the regulation of tomato pericarp thickness and fruit size [14]. The highest concentration of lignin was detected in the pericarp cell wall of the *GP12* inbred line, with extremely high popping expansion [15]. Based on these results, we proposed the scientific hypothesis that the regulatory mechanism of pericarp thickness and components is related to specific transcription factors. Hence, screening new transcription factors related to pericarp thickness, creating new germplasm with high-quality pericarp traits and cultivating new varieties of macadamia nuts are effective ways to address the needs of the industry both in China and globally.

## 2. Results

### DEGs in the Three Macadamia Varieties with Different Pericarp Thicknesses

**Figure 1:**
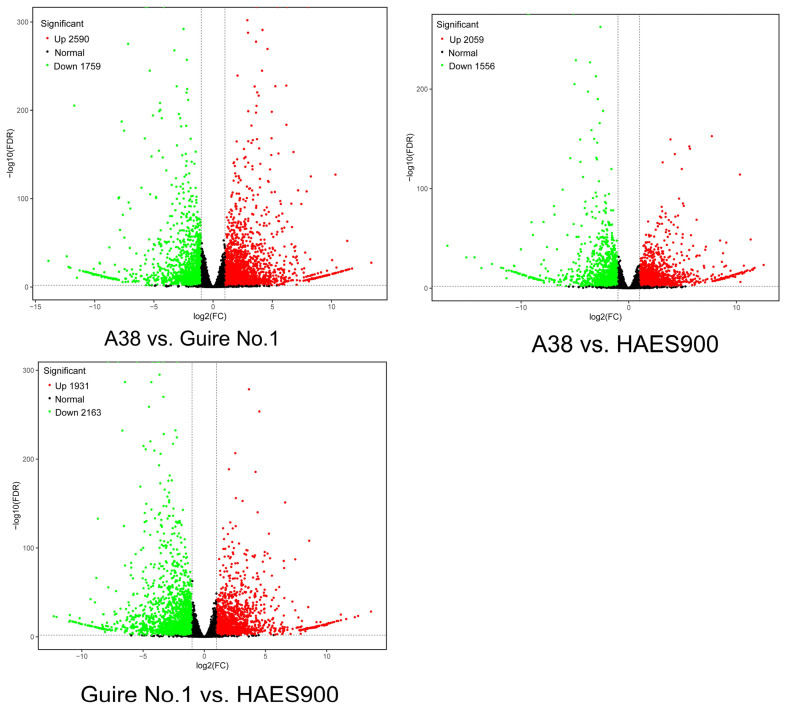
Volcano map of DEGs in the 3 macadamia varieties with different pericarp thicknesses: A38, Guire No.1, and HAES900.

A transcriptome analysis of nine samples from the three macadamia varieties with different pericarp thicknesses (A38, Guire No.1, and HAES900), and three repeats, was completed. A total of 61.30 Gb of clean data was obtained, and the clean data of each sample reached 6.07 Gb, while the Q30 base percentage was 94.00% or above. The clean reads of each sample were compared with the specified reference genome, and the comparison efficiency ranged from 87.52% to 90.94% (Supplementary Table S1). Based on the comparative results, an alternative splicing prediction analysis, a gene structure optimization analysis, and new gene discovery were carried out, and 3837 new genes were discovered, of which 1532 were functionally annotated. As can be seen from Figure 1, the number of differentially expressed genes (DEGs) in the transcriptome analysis of pericarp between the three varieties reached a significant level. The total number of differential genes between A38 and Guire No.1 was 4349, of which 2590 were upregulated and 1759 downregulated. The total number of DEGs between A38 and HAES900 was 3615, of which 2059 were upregulated and 1556 were downregulated. The total number of DEGs between Guire No.1 and HAES900 was 4094, of which 1931 were upregulated and 2163 downregulated (Figure 1).

**Figure 2:**
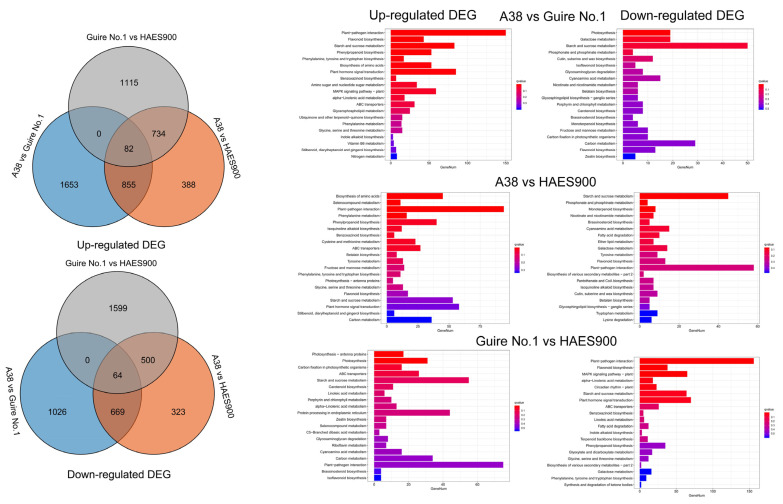
Upregulated and downregulated DEGs in the three macadamia varieties with different pericarp thicknesses: A38, Guire No.1, and HAES900.

As shown in Figure 2, the upregulated differential expression genes between A38 and Guire No.1 were mainly in flavonoid biosynthesis, phenylpropanoid biosynthesis, and phenylalanine, tyrosine, and tryptophan biosynthesis, and the gene ratio was 4.53%, 5.58%, and 1.79%, respectively. The downregulated differential expression genes between A38 and Guire No.1 were mainly in photosynthesis and cutin, suberine, and wax biosynthesis, and the gene ratio was 3.23% and 2.04%, respectively. The upregulated differential expression genes between A38 and HAES900 were mainly in phenylpropanoid biosynthesis, phenylalanine, tyrosine, and tryptophan biosynthesis, and the flavonoid biosynthesis pathways, and the gene ratio was 5.33%; 1.47%; and 2.27%, respectively. The downregulated differential expression genes between A38 and HAES900 were mainly in fatty acid degradation, flavonoid biosynthesis, and cutin, suberine, and wax biosynthesis pathways, and the gene ratio was 1.98%, 2.58%, and 1.79%, respectively. The upregulated differential expression genes between Guire No.1 and HAES900 were mainly in photosynthesis—antenna proteins, photosynthesis, and carbon fixation in photosynthetic organisms, and the gene ratio was 2.40%, 4.38%, and 2.26%, respectively. The downregulated differential expression genes between Guire No.1 and HAES900 were mainly in phenylpropanoid biosynthesis and phenylalanine, tyrosine, and tryptophan biosynthesis, and the gene ratio was 4.41% and 1.13%, respectively (Supplementary Table S2).

**Figure 3:**
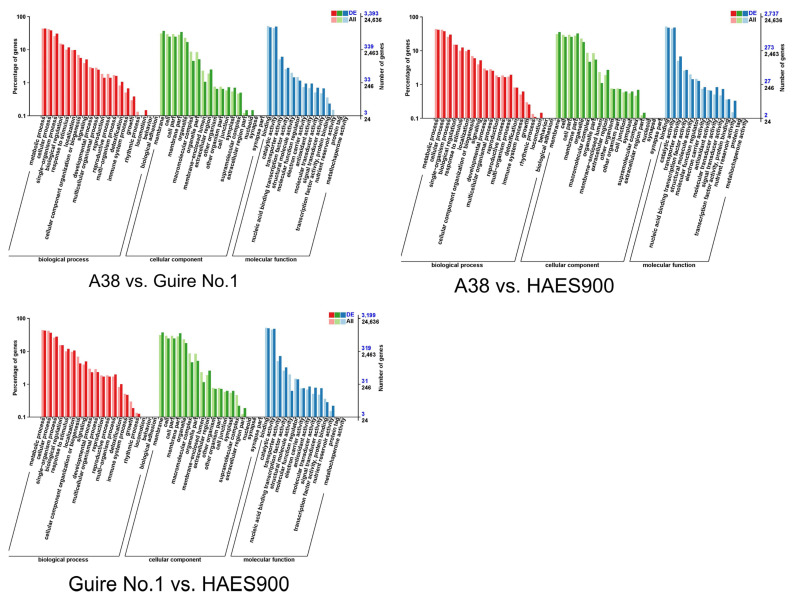
GO analysis of DEGs in the three macadamia varieties with different pericarp thicknesses: A38, Guire No.1, and HAES900.

The COG (Cluster of Orthologous Groups of proteins) database enables the orthologous classification of gene products. As shown in Figure 3 and Supplementary Table S3, the most significant differences in the pathway between A38, Guire No.1, and HAES900 are within three main branches: biological process, molecular function, and cellular component. For molecular function, transcription factor activity, protein binding, and nucleic acid binding transcription factor activity have a high DEG ratio. To further explore the key transcription factors in pericarp thickness and nutrition composition, the differentially expressed transcription factors were analyzed (Supplementary Table S4), and the MYB, bHLH, and NAC transcription factor families were the main differentially expressed gene families. Furthermore, all upregulated and downregulated genes were comprehensively analyzed to find potential target genes of MYB, bHLH, and NAC transcription factor (Supplementary Tables S5 and S6). 

**Figure 4:**
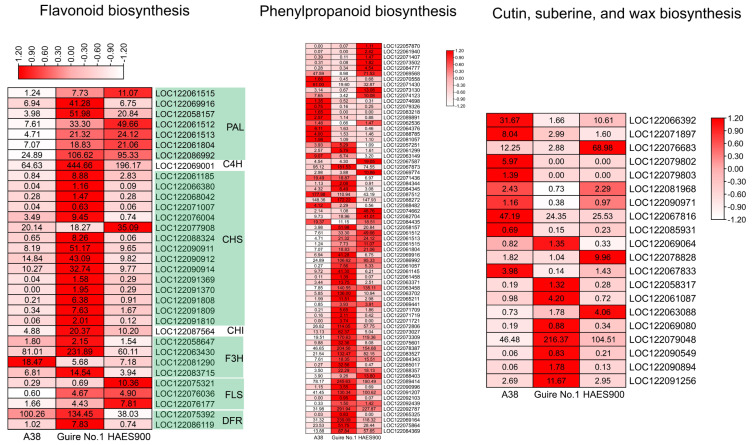
DEG expression of flavonoid, phenylpropanoid biosynthesis, and cutin, suberine, and wax biosynthesis in the three macadamia varieties with different pericarp thicknesses: A38, Guire No.1, and HAES900.

As shown in Figure 4, for flavonoid biosynthesis-related differentially expressed genes, most of which were highly expressed in Guire No.1, the FPKM of the most highly expressed PAL, C4H, F3H, and DFR was 106.62, 444.66, 231.89, and 134.45, respectively (Supplementary Table S7). For phenylpropanoid biosynthesis-related differentially expressed enzyme genes, most of which were highly expressed in Guire No.1, the FPKM of LOC122092787, LOC122089414, LOC122069164, LOC122078387, LOC122067873, LOC122088272, LOC122073309, and LOC122063458 was 291.94, 245.63, 239.09, 204.56, 181.53, 172.22, 170.63, and 140.55, significantly higher than that in A38 and HAES900 (Supplementary Table S8). Most of the cutin, suberine, and wax biosynthesis-related differentially expressed genes were highly expressed in Guire No.1. The FPKM of LOC122066392, LOC122081968, and LOC122071897 was 216.37, 24.35, and 11.67, respectively (Supplementary Table S9). 

**Figure 5:**
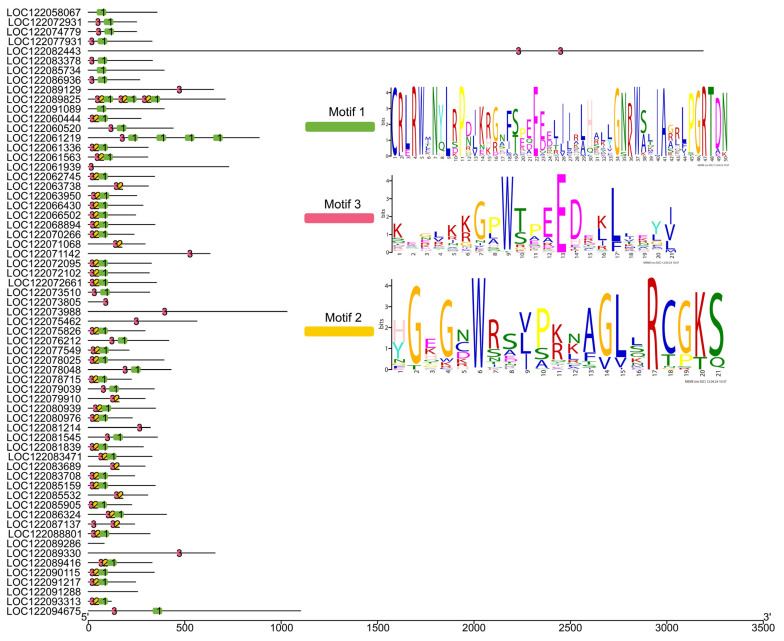
Protein structures of 63 MiMYBs for transcriptome analysis in the three macadamia varieties with different pericarp thicknesses: A38, Guire No.1, and HAES900. Green, yellow and pink color box mean Motif 1, Motif 2 and motif 3, respectively.

As shown in Figure 5, a total of 63 MiMYB TFs were identified in this study. According to their motif and protein structures, four 1R-MYB, fifty-six 2R-MYB, one 3R-MYB, and two 4R-MYB transcription factors were classified (Supplementary Table S10). Three specific motifs were found and used as the main indices for classification. 

**Figure 6:**
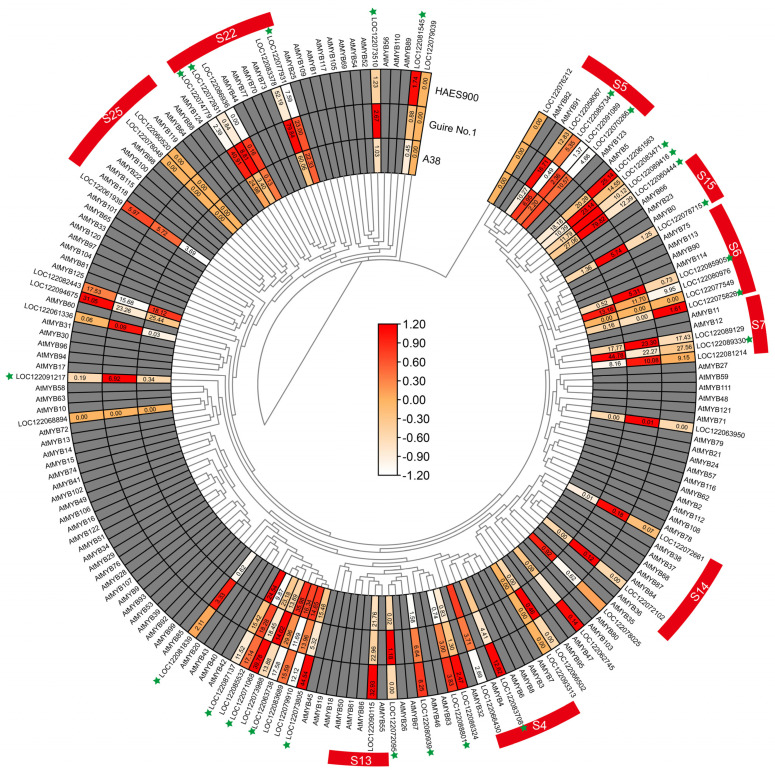
Phylogenetic and expression analysis of 56 R2R3-MiMYBs in the three macadamia varieties with different pericarp thicknesses: A38, Guire No.1, and HAES900. * means significant difference level (*p* < 0.05).

Furthermore, a phylogenetic analysis of 56 R2R3-MiMYBs compared with those of Arabidopsis was conducted; as shown in Figure 6, the 56 R2R3-MiMYBs can be divided into nine subgroups, namely, S4, S5, S6, S7, S13, S14, S15, S22, and S25 (Supplementary Table S10). Of these, S4, S6, and S7 participate in flavonoid biosynthesis. In the S4 subgroup, LOC122066430 and LOC122083708 were the homologous genes of AtMYB4 and AtMYB6 [16]. In the S6 subgroup, LOC122085905 and LOC122080976 were the homologous genes of AtMYB114 and AtMYB90 [17,18]. In the S7 subgroup, LOC122089129 and LOC122089330 were the homologous genes of AtMYB11 and AtMYB12 [19,20]. 

**Figure 7:**
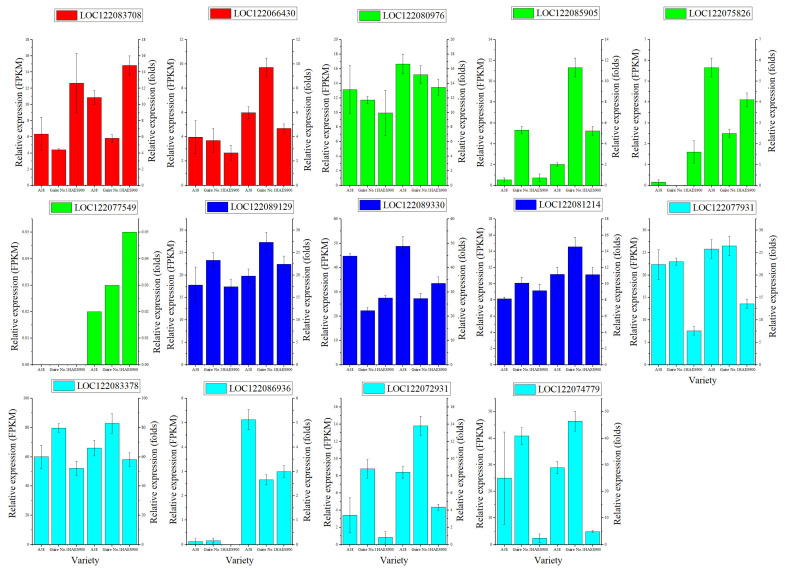
RT-qPCR verification for 14 *MiMYBs*’ gene. In each bar graph, the left was the data from transcriptome analysis (FPKM), and the right was the RT-qPCR results (folds). Red, S4 subgroup; Green, S6 subgroup; Blue, S7 subgroup; Cyan, S22 subgroup.

Furthermore, 14 MiMYBs’ gene expression level from S4, S6, S7, and S22 subgroups were verified by RT-qPCR method (Supplement Table S12). As shown in Figure 7, the fluorescence quantitative results were consistent with those of transcriptome analysis.

## 3. Discussion

With the continuous innovation and expansion of genetics, breeding, and food science and technology, studies on the content and antioxidant activity of different solvent extracts of macadamia pericarp have been carried out at home and abroad, and obvious differences have been identified in the contents of total phenols, total flavonoids, tannins, and total antioxidant capacity in macadamia pericarps [21,22]. A genomic analysis of Guire No.1 showed that the macadamia genome has 14 chromosomes, encoding 37,728 genes [23]. 

In this study, the main innovative finding is that the S4, S6, and S7 MiMYB subgroups participate in flavonoid biosynthesis, which is related to pericarp thickness and nutrition composition. The MYB protein family is a large and functionally diverse family that is expressed in all eukaryotes [24]. The MYB protein has a highly conserved DNA-binding domain, which typically consists of four incomplete amino acid repeats (R), each of which has approximately 52 amino acids, and each repeat forms three α helixes. The second and third helixes of each repeat form a helix–turn–helix (HTH) structure with three regularly spaced tryptophan (or hydrophobic) residues, forming a hydrophobic core in a three-dimensional HTH structure [25]. MYB proteins can be classified into different classes based on the number of adjacent domain repeats (1, 2, 3, or 4): R1/R2-MYB, R2R3-MYB, R1R2R3-MYB, and 4R-MYB [26,27]. MYB plays an important role in regulating plant cell growth and development, abiotic stress and biotic stress, and metabolic reactions. The regulatory role of MYB family members in plant fruit traits mainly includes the direct regulation of pericarp thickness and the regulation of the active ingredients in fruit [28,29,30]. Based on a genome study of the Guire No.1 variety [23], a transcriptome analysis was performed of macadamia nut varieties A38, Guire No.1, and HAES900, with significant differences in pericarp thickness. A total of 63 MYB family members were found in the transcriptome (Figure 5 and Figure 6). We further found that the S4, S6, and S7 MiMYB subgroups participate in flavonoid biosynthesis. LOC122066430 and LOC122083708 are homologous genes of AtMYB4 and AtMYB6, etc. These are new findings which can be supported by previous studies. In a joint study of transcriptome and miRNA in maize pericarp thickness, it was found that miRNA164, miRNA167, and miRNA156 regulated miRNA–mRNA pairs that were involved in regulating pericarp thickness. miRNA164 regulates MYB transcription factors [5]. A transcriptome analysis revealed a maximum of 64 MYB differential genes, suggesting that MYB may directly regulate peel thickness [5]. On the other hand, MYB is a key regulatory gene for active ingredients in the peel. For example, MYB93 and MYB11 prevent damage to the peel from sunburn by modulating the biosynthesis of phenylpropionic acid and flavonoids [7]. AcMYB123 and AcbHLH42 in kiwifruit regulated the anthocyanin synthesis genes AcF3GT1 and AcANS during fruit and peel color change [13]. In the study of fruit acidity regulation, it was found that LcMYB5 in litchi regulates pH by regulating the acidification gene LcPH1 [31]. The spraying of gibberellin can brown the lychee peel, which is associated with LcMYB1 [32]. Phenols alter the thickness of corn peels and are associated with the c1/pl1/p1 Myb gene family of fumaric acid toxins [33]. A combined nuclear magnetic resonance (NMR) and transcriptome analysis showed that MYB was associated with proximal axial development [34]. In the study of the mechanism of peel protection, it was proved that rice *OsMYB102* and tomato *SlMYB102* transgenic *Arabidopsis thaliana* can improve antioxidant and senescence properties, and the mechanism is the MYB transcription factors directly regulating the expression of downstream SOS1 and *CYP707A3* genes [35,36]. The expression patterns of *MiMYBs* and their target genes in macadamia nuts with different pericarp thickness, light intensity, gibberellin, and other hormones also required analysis. This study reveals the transcriptional regulatory mechanism of macadamia pericarp flavonoid synthesis and provides a solid foundation for the screening of high-quality and high-yield macadamia nut germplasm resources.

## 4. Materials and Methods

### 4.1. Plant Materials

**Figure 8:**
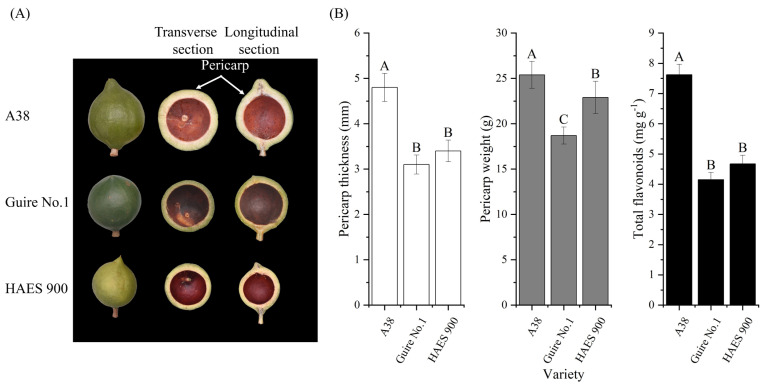
Phenotypes of the three macadamia varieties with different pericarp thicknesses—A38, Guire No.1, and HAES900: (**A**) phenotype; (**B**) pericarp thickness, weight, and total flavonoid content. Different uppercase letters mean extremely significant level (*p* < 0.01).

Three macadamia nut varieties, namely, A38, Guire No.1, and HAES900, with significant differences in pericarp size, were selected as study materials to determine their pericarp thickness, weight, and total flavonoid content (Figure 8). The fruit samples of the above cultivars were obtained from the macadamia germplasm resource nursery (E106°79′85″, N22°34′13″) of the Institute of Subtropical Agricultural Sciences in Guangxi, China. Usually, the full-blossom stage of the three macadamia nut varieties are from 24 to 27 March 2023. The fruit samples were collected at the same time, i.e., 120 days after pollination (dpp). The samples were collected in liquid nitrogen and stored in a −80 °C ultra-low-temperature freezer (Thermo Scientific, Waltham, MA, USA).

### 4.2. Methods

#### 4.2.1. Determination of Total Flavonoids

The total flavonoid content was determined using the method described by Benamar et al. (2010). An aliquot of 500 μL of pericarp extracts in methanol was mixed with 1500 μL of distilled water, followed by the addition of 150 μL of 5% NaNO_2_ (in water), and the mixture was allowed to react for 5 min. Following this, 150 μL of 10% AlCl_3_ (in water) was added and the mixture stood for a further 6 min. Finally, the reaction mixture was treated with 500 μL of 1 M NaOH (in water) and the absorbance at 510 nm was obtained against a blank prepared similarly, by replacing the extract with methanol using a spectrophotometer (YoukeL6, Shanghai, China). Total flavonoid content was calculated from a calibration curve, using catechin (5–25 μg mL^−1^) as the standard, and expressed as mg of catechin equivalents g^−1^ of lyophilized pericarp extracts. The experiment was performed in triplicate [37].

#### 4.2.2. RNA Extraction and RNA-Seq Analysis

The total RNA in the macadamia nut pericarp was extracted using an RNAprep Pure Plant Plus Kit (Tiangen Biotech, Beijing, China). The pericarp samples were subjected to RNA-seq with Biomarker technologies. Three biological replicates were carried out. RNA was extracted from each sample, and the concentration and integrity of each RNA sample were examined using NanoDrop (Thermo Scientific, Waltham, MA, USA), Qubit 2.0 (Invitrogen, Carlsbad, CA, USA), Agilent 2100 (Agilent, Palo Alto, CA, USA), etc. Only RNA with good quality could move on to the following procedures. Qualified RNA samples were processed for library construction. In order to ensure the quality of the library, Qubit 2.0 and Agilent 2100 were used to examine the concentration of cDNA and insert size. qPCR was performed to obtain a more accurate library concentration. A library with a concentration larger than 2 nM was acceptable. The qualified library was pooled based on pre-designed target data volume and then sequenced on an Illumina sequencing platform. Clean data with high quality were obtained by filtering raw data, which removed adapter sequences and reads with low quality. These clean data were further mapped to pre-defined reference genome-generating mapped data. Assessments of insert size and sequencing randomness were performed on the mapped data as library quality control. A basic analysis of the mapped data included gene expression quantification, alternative splicing analysis, novel gene prediction, and gene structure optimization. DEGs with a fold change ≥ 1.5 and a *p*-value < 0.05 were selected to analyze GO and KEGG enrichment. In order to understand the GO entries that were significantly enriched compared with the whole genomic background, clusterProfiler was used to conduct an enrichment analysis of biological processes, molecular functions, and cell components by using hypergeometric testing methods for the differential gene sets of each group. The term obtained from the enrichment results was visualized with a histogram. The function of different genes in this group could be predicted based on the information of GO functional enrichment (Supplementary Table S11) [38]. The filtered sequences were aligned to the reference genome and the transcriptome sequence data were assembled using StringTie software v2.2.0 [39]. The transcription factors were identified based on gene annotation in the database and the PlantTFDB platform v5.0 https://planttfdb.gao-lab.org/tf.php?sp=Mtr&did=Medtr1g086510.1 (20 March 2024) [40]. Using Muscle v5 [41] for multiple sequence alignment, IQ-TREE software v2.3.4 [42] was employed to construct a phylogenetic tree based on the maximum likelihood method. FigTree v1.4.4 was used to adjust the constructed phylogenetic tree. 

#### 4.2.3. cDNA Synthesis and RT-qPCR Verification

First, 1 µg of the sample was reverse-transcribed to cDNA with RevertAid Reverse Transcriptase (Thermo Scientific, Waltham, MA, USA) as follows: (1) Oligo(dT)^18^ primer at 1 µL and total RNA at 1 ng were added with water to 12 µL, and the reaction was run at 65 °C for 5 min. (2) The following components were added in the indicated order: 5× Reaction Buffer at 4 µL, RiboLock RNase Inhibitor (20 U/µL) at 1 µL, 10 mM dNTP Mix at 2 µL, and RevertAid M-MuLV RT (200 U/µL) at 1 µL for a total volume of 20 µL; the reaction was run at 42 °C for 60 min, and then at 70 °C for 5 min. RT-qPCR was conducted using CFX96 (Bio-Rad, Hercules, CA, USA) according to the following steps: 8.2 µL of ddH2O, 0.4 µL of forward primer, 0.4 µL of reverse primer, 10.4 µL of cDNA, and 2 × 10 µL of ChamQ Universal SYBR qPCR Master Mix (Vazyme, Nanjing, China) were added into one reaction solution with real-time PCR. The following reaction steps were used: 95 °C for 30 s to denature; 5 °C for 10 s and 60 °C for 30 s, up to 40 cycles; 95 °C for 15 s, 60 °C for 60 s, and 95 °C for 15 s. MiActin was used as a reference gene (HQ260674.1) (Supplement Table S12). Three biological replicates were performed for each sample. Expression levels were calculated using 2^−∆∆Ct^. Three biological repeats and three experimental replicates were carried out for each sample [38].

## Data Availability

All raw data can be found on the website “http://www.ncbi.nlm.nih.gov/bioproject/1091847 (26 March 2024)”, the BioProject ID: PRJNA1091847.

## Supplementary Materials

The following supporting information can be downloaded at https://www.mdpi.com/article/10.3390/ijms25136840/s1.
